# Molecular insights into the inhibitory potential of anthocyanidins on glucokinase regulatory protein

**DOI:** 10.1371/journal.pone.0288810

**Published:** 2023-07-19

**Authors:** Christian Kenneth, Daru Seto Bagus Anugrah, Jeffry Julianus, Sendy Junedi

**Affiliations:** 1 Biotechnology Study Program, Faculty of Biotechnology, Atma Jaya Catholic University of Indonesia, Jakarta, Indonesia; 2 Faculty of Pharmacy, Sanata Dharma University, Yogyakarta, Indonesia; 3 Faculty of Biotechnology, Universitas Atma Jaya Yogyakarta, Yogyakarta, Indonesia; Universiti Teknologi Malaysia, MALAYSIA

## Abstract

Computational methods were used to investigate six anthocyanidins exhibiting antidiabetic activity by inhibiting glucokinase regulatory protein (GKRP) activity. Density functional theory was used to optimise the geometry of anthocyanidins and calculate their quantum chemical properties. A blind docking method was employed to conduct a molecular docking study, which revealed that delphinidin (Del), cyanidin (Cya), and pelargonidin (Pel) as potential GKRP inhibitors with the lowest binding free energy of -8.7, -8.6, and -8.6 kcal/mol, corresponding to high binding affinity. The molecular dynamics study further verified the blind docking results by showing high GKRP-F1P complex stability and high binding affinity calculated through the MM/GBSA method, upon the binding of pelargonidin. The lower RMSF values of pivotal GK-interacting residues for GKRP-F1P-Pel compared to GKRP-F1P, as a positive control, indicating pelargonidin ability to maintain the inactive conformation of GKRP through the inhibition of GK binding. The key residues that control the binding of the F1P to GKRP and anthocyanidin to GKRP-F1P were also identified in this study. Altogether, pelargonidin is anthocyanidins-derived natural products that have the most potential to act as inhibitors of GKRP and as antidiabetic nutraceuticals.

## Introduction

Type 2 diabetes mellitus (T2DM) is the most prevalent type of diabetes, with around 90% of incidents worldwide [1]. Commonly used synthetic antidiabetic drugs, such as metformin, sulphonylureas, and thiazolidinediones, are prone to cause several side effects, such as nerve damage, increased risk of cardiovascular disease, and even bladder cancer, with prolonged use [2]. Thus, investigations of natural products that possess antidiabetic activity are conducted, especially anthocyanins.

Anthocyanins are abundant flavonoid pigments found in flowers, fruits, and sometimes leaves [3]. Several anthocyanins are typically used as food additives or naturally consist in food products, such as cyanidin-3-glucoside, delphinidin-3-glucoside, malvidin-3-glucoside, pelargonidin-3-glucoside, peonidin-3-glucoside, and petunidin-3-glucoside [4, 5]. After being absorbed into the gastrointestinal wall, anthocyanins undergo hydrolysis into aglycones by β-glucosidase in hepatocytes [3, 5]. The hydrolysis products, anthocyanidins, are prominent for their antidiabetic activity by inhibiting the activity of α-amylase and α-glucosidase, which has a critical function in degrading polysaccharides into monosaccharides, such as glucose. As a result, blood glucose levels will decrease [6].

Another recent potential target for developing antidiabetic agents that are actively studied is hepatocytes glucokinase (GK). GK is one of the predominant enzymes involved in glucose homeostasis, catalysing the transfer of the phosphate group into glucose to create glucose 6-phosphate (G6P). Hence, activation of GK leads to an increase in the rate of blood glucose transfer into hepatocytes’ cytoplasm. However, preliminary reports demonstrate that hyperactivation of GK can increase the risk of hypoglycemia [7]. For this reason, investigations of GK activators have shifted to its regulatory protein, GKRP. GKRP regulates GK activity by establishing the GK-GKRP complex, causing GK to be inactive [8]. Antidiabetic agents that can bind to GKRP may induce a prolonged activity of GK, thereby reducing blood glucose levels. As natural products that exhibit antidiabetic activity, anthocyanidins have not been studied using this novel mechanism.

**Fig 1:**
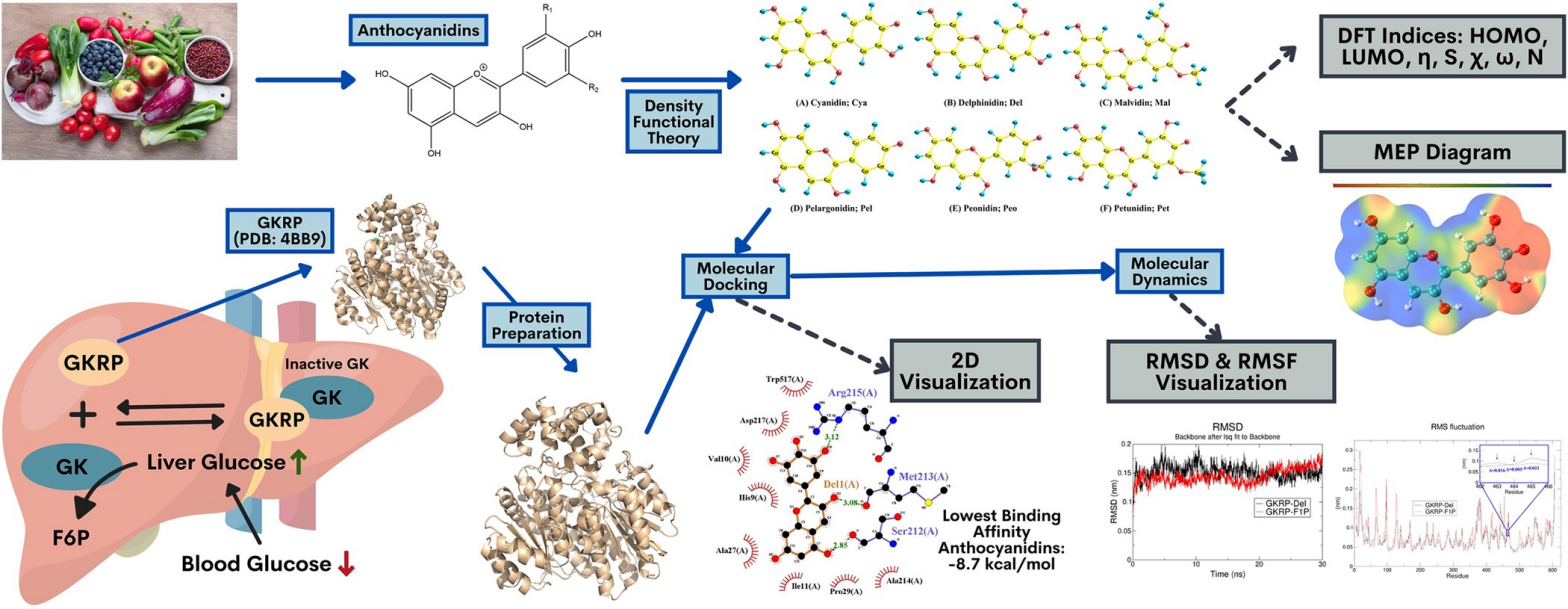
Illustration in silico method to analyse the antidiabetic activity of anthocyanidins against GKRP.

The advances of computational technology in biotechnology and quantum chemistry have enabled a theoretical approach of elucidating and simulating intermolecular and even interatomic interactions. Accordingly, this research will be done using in silico method, including density functional theory (DFT), molecular docking, and molecular dynamics (MD) to analyse the antidiabetic activity of anthocyanidins against GKRP (**Fig 1**). In addition, the quantum chemical properties and action mechanism of the ligand can be elucidated through the interaction between GKRP-anthocyanidin complex, allowing for the development of novel antidiabetic agents.

## Methods

### Density functional theory (DFT) calculations

The SDF structures of 6 screened anthocyanidins were downloaded from PubChem (https://pub-chem.ncbi.nlm.nih.gov/) and deprotonated according to the pH of intracellular hepatocyte, 7, using ChemDraw. Then, each geometry of the ligands was optimised by NWChem [9] with the density functional theory (DFT) method using the B3LYP functional and the 6-311G(d, p) basis set. The conceptual DFT indices, including E_HOMO_, E_LUMO_, chemical hardness (η), chemical softness (S), electronegativity (χ), electrophilicity (ω), and nucleophilicity (N) were calculated to elucidate the quantum chemical properties of anthocyanidins [10]. The HOMO-LUMO diagram was visualised with GaussView 6 [11], while the molecular electrostatic potential (MEP) was generated with Multiwfn [12] and visualised with VMD [13] to analyse the putative regions within each anthocyanidin that favours electrostatic-induced intermolecular interactions.

### Molecular docking studies

The structure of *Homo sapiens* GKRP (PDB ID: 4BB9) was retrieved from RCSB PDB (https://www.rcsb.org/) with the resolution 1.47 Å. The open-source version of PyMOL was used to extract the F1P ligand as a positive control [6], while the calcium ion and the water molecules were removed. The missing residues in the loop conformation between Glu63 and Ser69 were added, optimised with ModLoop Server [14, 15], and subsequently, minimised by Yasara Energy Minimization Server [16]. The Yasara Energy Minimization Server was used for its ability to perform multi-step optimisation using the two-step steepest descent method, where the first step is to tune the predicted side-chain rotamers using an implicit solvent, and the second step is to perform a steepest descent followed by a simulated annealing minimization to eliminate collisions derived from the cis-peptide bond and wrong side-chain isomers [16], leading to an increase in favored GKRP residues based on the Ramachandran plot **(S1 Table)**. The results indicate that the Yasara Energy Minimization (Yasara-EM) yielded a higher value for the “Favored” category compared to Gromacs Energy Minimization (Gromacs-EM), with respective values of 92.9% and 89.3%. Prior to molecular docking, all polar hydrogen atoms were added, and Gasteiger charges were calculated for GKRP and the optimised anthocyanidins structure with AutoDockTools-1.5.6 from the MGLTools software suite [17]. The blind docking method was used to discover possible ligand-binding sites outside the GKRP active site. QuickVina-W [18] was utilised to perform the calculations with the advantages of the inter-process spatio-temporal integration method and Vina scoring function in a 76 × 80 × 66 Å^3^ grid box covering the whole GKRP structure. Furthermore, the binding site and intermolecular interactions occurred between the lowest energy pose of each anthocyanidin, and GKRP was used and analysed using the Protein Imager [19] and Discovery Studio Visualizer [20].

### Molecular dynamics studies

After molecular docking study, each anthocyanidin was subjected to multi-ligand molecular dynamics (MD) simulation together with F1P using GROMACS 2021.2 [21] to examine the ligand-receptor interactions comprehensively and the behavior of GKRP in GKRP-F1P and GKRP-F1P-anthocyanidin complexes. The topology of GKRP was prepared with AMBER99SB-ILDN all-atom forcefield [22] and TIP3P water model, while the ligand topology, both F1P and anthocyanidins, with GAFF2 [23], was obtained using ACPYPE [24]. The simulation system is a dodecahedron box neutralized with randomly added 7 Na^+^ and a total of 17464 water molecules. After performing energy minimisation, the system equilibrated with 100 ps NVT and NPT ensemble, followed by MD simulation at 310 K, 1 bar, and 0.002 ps timestep. Hydrogen bonds were constrained using the LINCS algorithm and long distance electrostatics were calculated using the Particle Mesh Ewald (PME) method. The modified Berendsen thermostat was used for the temperature coupling scheme and the Parinello-Rahman method for the pressure coupling scheme. MD simulations were run three times for each system (triplicates) with multiple 5 ns window sizes later concatenated with *gmx trjcat*, each resulting a 100 ns simulated system.

MD trajectories analysis performed using the GROMACS built-in tools to generate multiple parameters, such as root-mean-square deviation (RMSD), root-mean-square fluctuation (RMSF), radius of gyration (Rg), and solvent accessible surface area (SASA). Cluster analysis of the GKRP backbone RMS was also determined using *gmx cluster* with a cutoff calculated from the RMSD end-to-end distance divided by two while using *gmx sham* to estimate the free energy landscape created from RMSD and Rg values. Matplotlib [25], Seaborn [26], and Plotly [27] are Python libraries that were used to create and visualize the plots.

### Binding free energy calculation and per-residue decomposition analysis

Molecular mechanics Generalized Born surface area (MM/GBSA) method was employed to estimate the relative binding free energy of the four final protein-ligand complexes and the energy contribution per residue to the binding energy. In combination with GROMACS, gmx_MMPBSA tool [28, 29] was utilised to perform this analysis. GB model used was GB-Neck which offers a correction term from the previously developed GB model GB-OBC, leading to an increase in the accuracy of determination of binding free energies [30]. Pairwise decomposition energy screening was performed for GKRP’s residues located within 5 Å of each ligand binding site. The results of this study are then visualised using two Python statistical graphics libraries: Matplotlib [25] and Seaborn [26].

## Result and discussion

**Fig 2:**
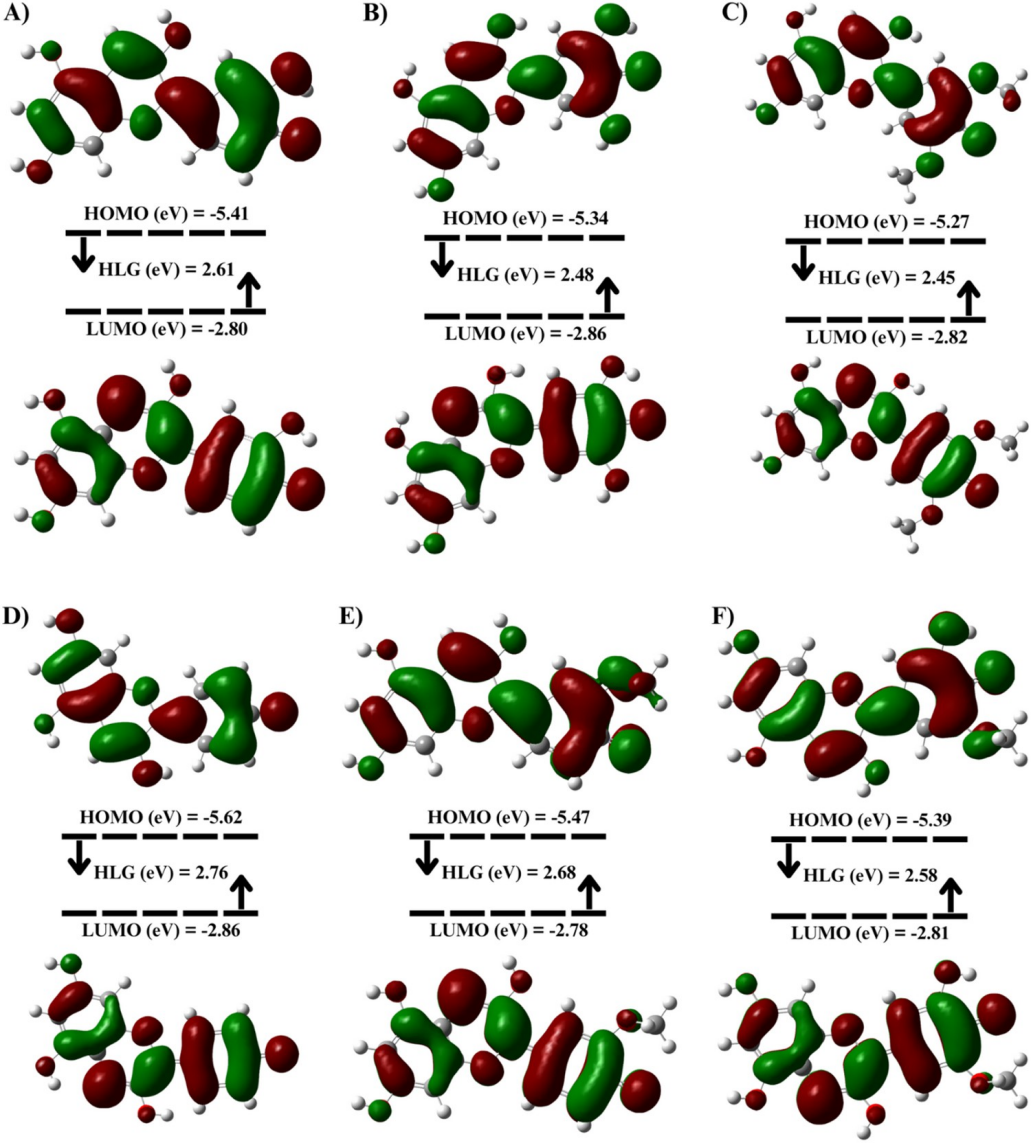
HOMO-LUMO diagram of anthocyanidins at pH 7: **(A)** Cya, **(B)** Del, **(C)** Mal, **(D)** Pel, **(E)** Peo, and **(F)** Pet with HOMO-LUMO gap (HLG) depicted with GaussView 6.

Six anthocyanidins were selected as studied compounds: cyanidin (Cya), delphinidin (Del), malvidin (Mal), pelargonidin (Pel), peonidin (Peo), and petunidin (Pet). DFT-optimized structure of anthocyanidins at pH 7 is used in the calculation of quantum chemistry parameters, and the results are shown in **Fig 2** and **Table 1**. Through conceptual DFT indices study [10, 31], the intrinsic bioreactivity of anthocyanidins is emphasised. Highest occupied molecular orbital (HOMO) energy represents the ability of a molecule to act as an electron donor. In contrast, lowest unoccupied molecular orbital (LUMO) energy entitles the capacity of a molecule to accept an electron. The red regions of the molecules depicted in **Fig 2** indicate high electron density, while green regions indicate the opposite. Parr (1995) termed the gap between HOMO-LUMO energy as a chemical hardness and the inverse as a chemical softness, which portrays the eagerness of a molecule to resist and accept the exchange of electron density, respectively [10, 32]. Optimised anthocyanidins demonstrate greater hardness values over softness, with Pel being the hardest, indicating a high tendency to resist polarisation and remain stable, while Mal being the softest, indicating the most chemically reactive compound among the anthocyanidins.

**Table 1:** Conceptual DFT indices calculation of anthocyanidins; HOMO & LUMO energy, chemical hardness (η), chemical softness (S), electronegativity (χ), electrophilicity (ω), and nucleophilicity index (N).

Parameters	E_HOMO_ (eV)	E_LUMO_ (eV)	η (eV)	S (eV^-1^)	χ (eV)	ω (eV)	N (eV)
**Definition**	–	–	$E_{L}-E_{H}$	$\frac{1}{\eta }$	$\frac{-(E_{H}+E_{L})}{2}$	$\frac{\chi^{2}}{2\eta }$	$\frac{E_{H}-{E_{H(TCE)}}^{}}{2}$ *
**Values**							
Cya	-5.41	-2.80	2.61	0.38	4.10	3.22	1.98
Del	-5.34	-2.86	2.48	0.40	4.10	3.39	2.01
Mal	-5.27	-2.82	2.45	0.41	4.05	3.35	2.05
Pel	-5.62	-2.86	2.76	0.36	4.24	3.26	1.87
Peo	-5.47	-2.78	2.68	0.37	4.13	3.17	1.95
Pet	-5.39	-2.81	2.58	0.39	4.10	3.26	1.99

*E_H(TCE)_ = -9.37 eV

The electrophilicity and nucleophilicity index encompasses the likelihood of an electrophile acquiring an extra amount of electron density and a nucleophile sharing electron density [10]. High electrophilicity values, increasing from Peo → Cya → Pel → Pet → Mal → Del, correlate with being strong electrophiles (ω > 1.5 eV) [10], implying anthocyanidins are prone to attack electron-density-rich species. In addition, anthocyanidins are marginal nucleophiles (N < 2.0), except for Del, 2.01, and Mal, 2.05, which are moderate nucleophiles (2.0 < N < 3.0) [10]. Therefore, Del and Mal are more likely to attack electrophilic regions than the other anthocyanidins.

**Fig 3:**
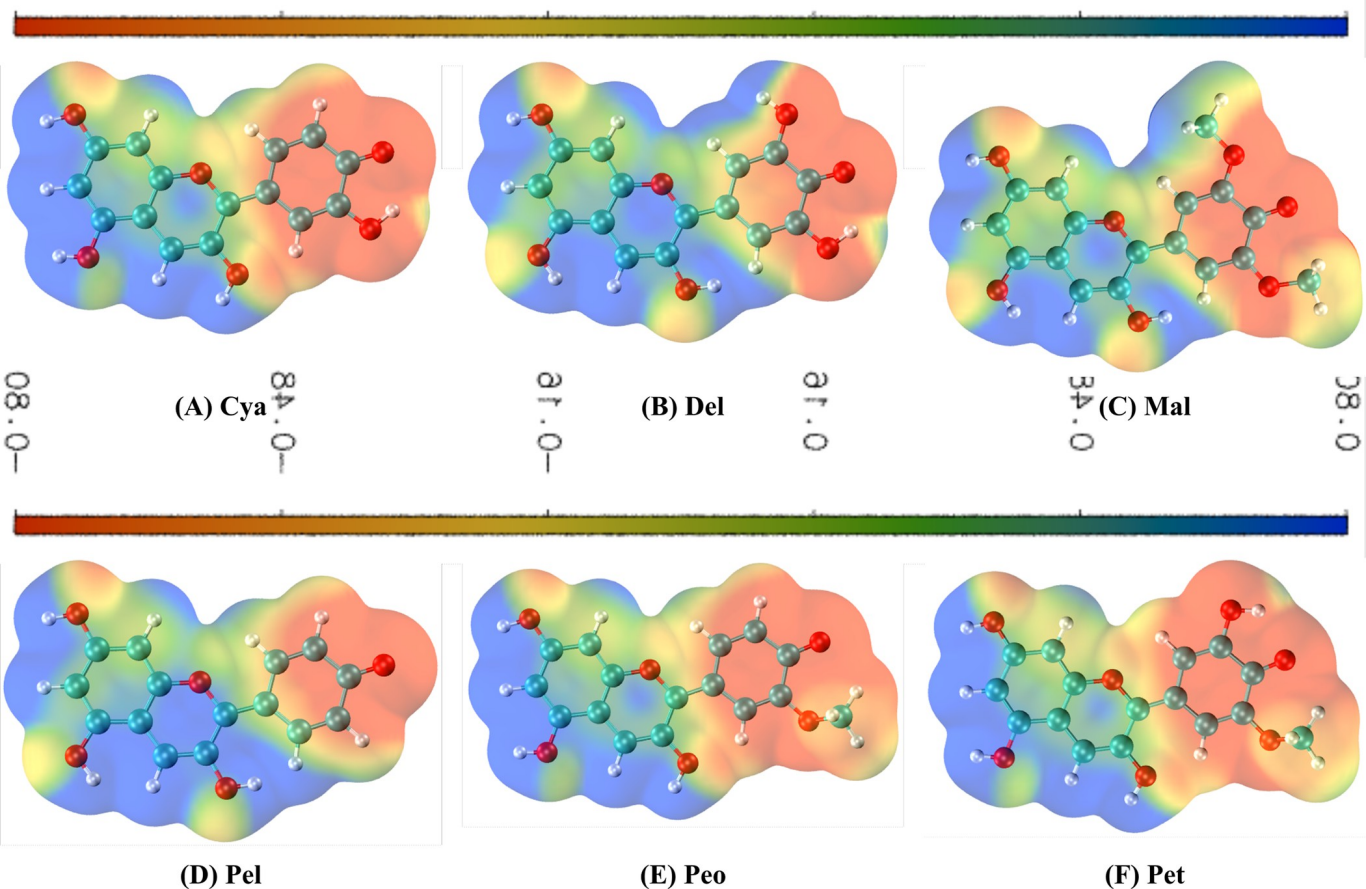
Molecular electrostatic potential (MEP) map of each anthocyanidin illustrated using VMD.

The electrostatic potential distribution of anthocyanidins depicted in **Fig 3** is essential for predicting the reactive sites within each anthocyanidin involved in intermolecular forces with the target molecule [32]. The positive electrostatic potential is increased from red → orange → yellow → green → blue. Oxygen atoms (O) attached to the benzene ring from each anthocyanidin are shown in red, indicating a high chance of being involved in a nucleophilic attack. However, O in the pyrylium ring is displayed in blue, implying a slight to substantial electron-deficient region (Pet to Pel) within the ring. Additionally, blue hydrogens (H) attached directly and indirectly (in the form of hydroxyl hydrogens) to the pyrylium-benzene ring have considerable potential for being targeted by nucleophiles, with green-coloured hydrogens, such as H-27 in Cya and Peo, H-29 in Del, H-30 in Mal and Pet, and H-25 in Pel, being more neutral.

QuickVina-W was employed for the blind docking calculations due to its advantages in terms of the inter-process spatio-temporal integration method [18]. In order to compare the performance of Autodock Vina and QuickVina in anthocyanidin-GKRP complexes, a comparison was conducted and the results are presented in **S2 Table**. The findings unequivocally showed that the calculations carried out with QuickVina-W consistently produce more stable outcomes compared to Autodock Vina across five repetitions. In accordance with the blind docking results presented in **Table 2**, the binding free energy of anthocyanidins ranging from -8.7 to -8.2 kcal/mol demonstrates an insignificant difference in blind docking results. Compared to F1P, which is used as a positive control due to its known regulatory action to inhibit GKRP-GK complex formation [6] with a binding free energy of -7.6 kcal/mol, all anthocyanidins show stronger binding to GKRP than F1P. Based on the three best compounds with the highest binding energy values of -8.7 kcal/mol and -8.6 kcal/mol, respectively, Del, Cya, and Pel, were selected for further studies.

**Table 2:** Blind docking statistics of anthocyanidins against GKRP sorted by the lowest binding free energy.

No.	Compound	Binding Free Energy (kcal/mol)
1	Del	-8.7
2	Cya	-8.6
3	Pel	-8.6
4	Peo	-8.4
5	Pet	-8.3
6	Mal	-8.2
Control	F1P	-7.6

**Fig 4:**
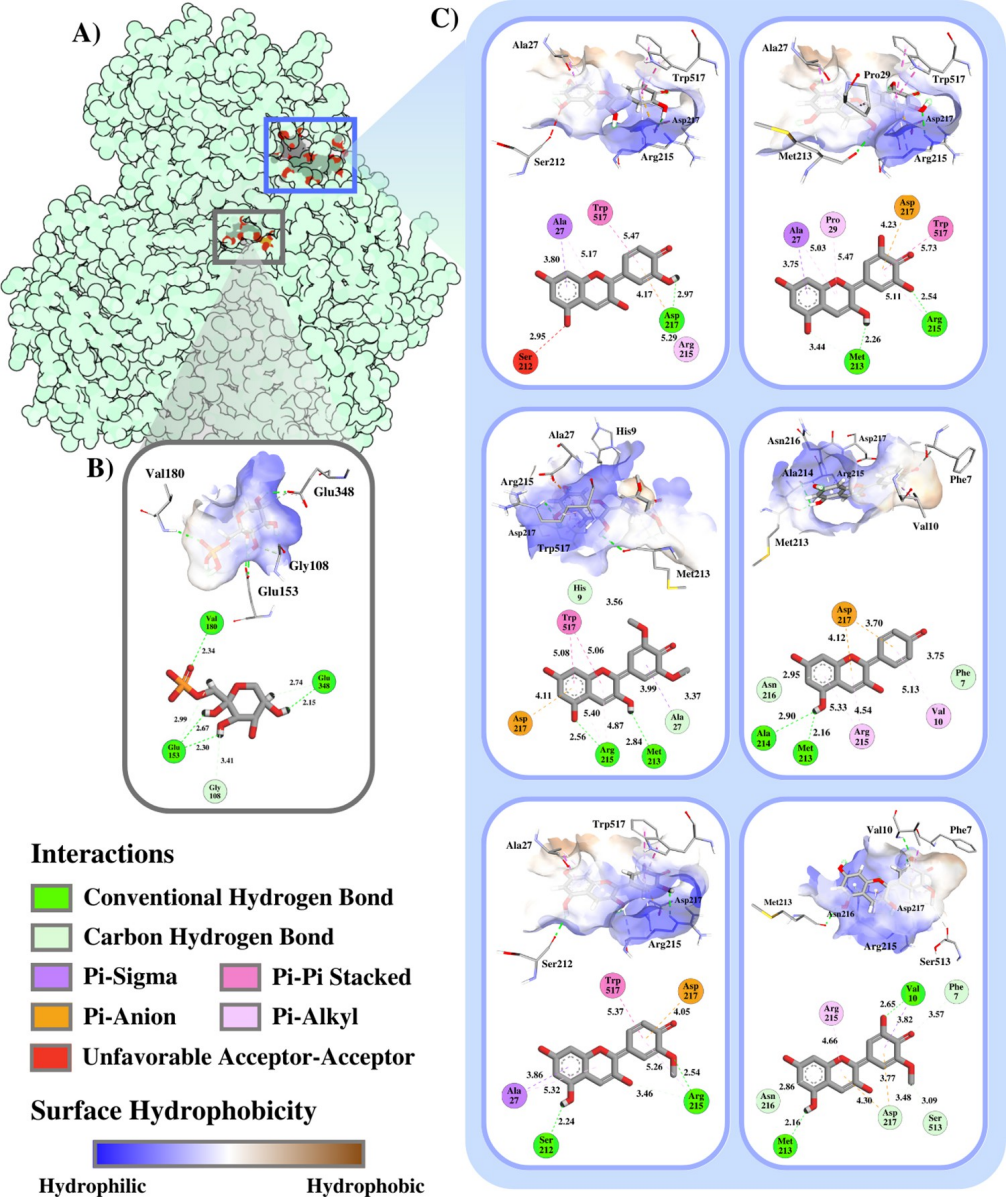
**(A)** The binding site of anthocyanidins and F1P within the GKRP, types and distance of non-covalent interactions with interacting residual surface hydrophobicity of **(B)** F1P-GKRP and **(C)** anthocyanidins-GKRP: first row from left: Cya and Del, second row from left: Mal and Pel, third row from left: Peo and Pet; are visualised with Protein Imager and Discovery Studio Visualizer.

In general, the binding site differs between F1P-GKRP and anthocyanidin-GKRP **(Fig 4A)**. However, both molecules are still located around the active site of GKRP, while F1P is located on the inside compared to the anthocyanidins on the outside (**S1 Fig**). This could be attributed to the low hydrophobicity of the F1P binding pocket, as indicated by a smaller brown region **(Fig 4B)**, in contrast to anthocyanins that possessed a more pronounced brown region **(Fig 4C)**. Moreover, F1P is composed of only single aromatic hydroxyl-rich pyranose ring, which possesses a smaller molecular size and higher hydrophilic than the anthocyanidins that feature three aromatic rings: pyrylium and pyrylium-benzene ring [33]. This makes it more difficult for the anthocyanidins to bind to the deeper, narrower active site of the GKRP.

Of all the amino acid residues that interact with anthocyanidins, as shown in **Fig 4C**, Arg215 and Asp217 are the most frequently interacting residues, meaning that these residues could play a crucial role in establishing the stable GKRP-anthocyanidin complexes. Del interaction with the active cavity of GKRP is stabilized by 3 hydrogen bonds formed with Arg215 (conventional hydrogen bond) and Met213 (both conventional and unconventional hydrogen bond). As illustrated in the MEP diagram **(Fig 3)**, the hydroxyl group at C-12 of Del is in the blue region, implying the hydroxyl hydrogen act as a hydrogen bond donor. Unlike C-12 hydroxyl groups, the hydroxyl group at C-21 of Del situated in the red region indicates the oxygen of hydroxyl that contributes to hydrogen bonding as a hydrogen bond acceptor. Also, several residues such as Ala27 (π-sigma and π-alkyl interactions), Pro29 (π-alkyl interaction), Trp517 (π-pi stacking), and Arg215 (π-alkyl interaction) are involved in hydrophobic interactions, and Asp217, which undergoes a π-anion electrostatic interaction, contributes to the overall stability of the GKRP-Del complex.

The interaction of Cya with the GKRP active site is stabilized by a conventional hydrogen bond formed with Asp217. As shown in the MEP plot **(Fig 3)**, the hydroxyl group at C-19 of Cya is highlighted in red, indicating that the hydroxyl hydrogen acts as a hydrogen bond acceptor. Besides hydrogen bonding, Asp217 also initiated an electrostatic π-anion interaction, while Arg215 initiated a hydrophobic π-alkyl interaction, both with the benzene ring of Cya. On the other hand, Pel initiated the most hydrogen bonding with GKRP among the top 3 compounds, that is 4 hydrogen bonds: Met213 & Ala214 (bifurcated conventional hydrogen bonds) and Phe7 & Asn216 (carbon hydrogen bonds). Correlated with **Fig 3**, the hydroxyl group at C-12 of Pel is in the blue area, indicating that the hydroxyl hydrogen acts as a hydrogen bond donor. However, both Arg215 and Asp217 each established two hydrophobic π-alkyl interactions and two electrostatic π-anion interactions.

A low number of hydrogen bonds made by the Cya-GKRP interaction, resulting in it having higher binding affinity than Del-GKRP. In addition, it can be seen from **Fig 4C** that Cya and Ser212 created an unfavorable interaction between hydrogen bond acceptors, making the interaction of Cya-GKRP unstable. In contrast to the Cya-GKRP complex interactions, the Pel-GKRP complex shows that a total of four hydrogen bonds are formed. However, the established conventional hydrogen bonds are bifurcated, meaning they are derived from the same hydrogen bond donor, H-22 in Cya. Feldblum and Arkin (2014) show through their study using isotope-edited Fourier transform infrared (FTIR) measurements and DFT calculations that the strength of bifurcated hydrogen bonds is 50–60% lower than the strength of canonical hydrogen bonds [34], implying that the conventional hydrogen bonding between Met213 and Ala214 with Pel contributed lesser affinity rather than the conventional hydrogen bonding in the Del-GKRP complex.

Conversely, all interactions between F1P and GKRP are hydrogen bonds: 4 conventional hydrogen bonds and 2 carbon hydrogen bonds. Furthermore, the distance of hydrogen bonds between F1P-GKRP interactions is relatively short, especially in conventional hydrogen bonding, 2.15–2.67 Å compared to anthocyanidin GKRPs. Relative to the distance of a predicted hydrogen bond, the shorter the length, the stronger and more stable the protein-ligand interaction [35]. However, the binding energy is lower than all anthocyanidins, possibly due to the lack of hydrophobic interactions between F1P-GKRP **(Fig 4B)**, which also help to stabilise the complex and contribute to the calculation of the binding energy using the Vina scoring method [36].

**Fig 5:**
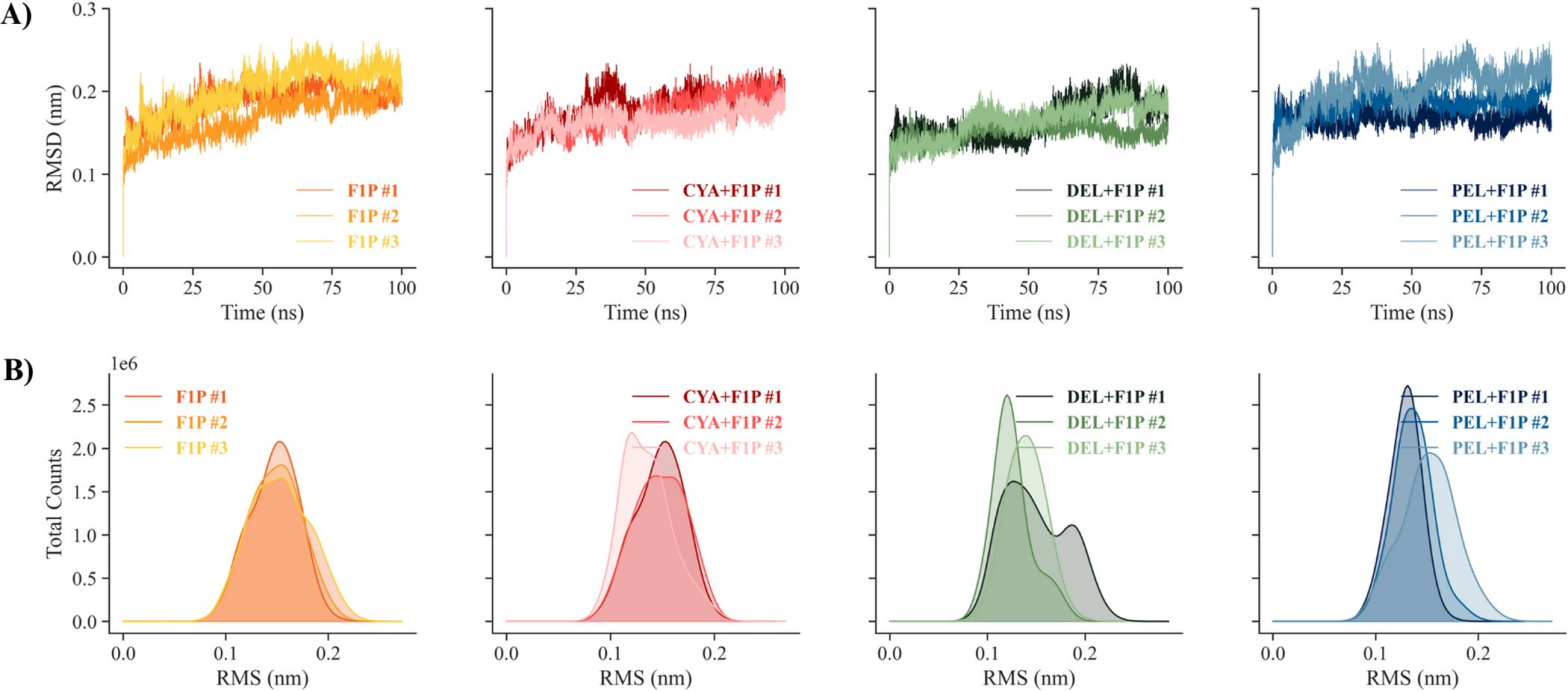
**(A)** RMSD and **(B)** cluster distribution of the GKRP backbone for the triplicates of the simulated GKRP-F1P and GKRP-F1P-anthocyanidin systems.

Furthermore, investigation of the prior top three anthocyanidins’ activity towards GKRP was uncovered by a triplicate in aquo system of molecular dynamics study. **Fig 5A** shows the RMSD trajectories of the GKRP backbone atoms to their respective initial coordinates observed for the interaction with its native ligand F1P, where the average RMSD values of the triplicates are the same and are 0.0839 nm. The same mean RMSD values of each triplicate of each system are also observed for the GKRP backbone of the GKRP-F1P-anthocyanidin complexes. This finding indicates that there is no significant difference in inactive GKRP backbone structure when anthocyanidins bind to the GKRP-F1P complex. However, there are distinct RMSD patterns where anthocyanidin bound to the GKRP-F1P complex more rapidly reaches a relatively stable region around 20 ns, while the native GKRP-F1P complex shows a notable increase from 0 ns to 55 ns and then starts remain stable up to 100 ns. This indicates that the binding of anthocyanidins leads to a faster establishment of the inactive GKRP form. However, DEL+F1P #1 and DEL+F1P #3 show different patterns where they remain stable for the first 50 ns and rise to 90 ns before falling and show a similar pattern and RMSD in the replicates.

A cluster analysis of the RMS distribution was performed to pinpoint the GKRP backbone structure distribution over a 100 ns time frame. Based on the **Fig 5B**, it can be stated that the RMS distribution of GKRP-F1P between replicates is more similar than the RMS distribution of GKRP-F1P-anthocyanidin between replicates. In addition, the mean RMS of GKRP-F1P and GKRP-F1P-Cya are similar and greater than the mean RMS of GKRP-F1P-Del and GKRP-F1P-Pel, both of which are also similar. This means that the dominant GKRP backbone structure caused by the binding of Del and Pel is more similar to the minimized structure. The difference that occurred between the replicates is caused by the chaotic nature of the dynamics of the molecular system, possibly pointing to a new GKRP backbone structure before it reaches the more stable conformation [37].

**Fig 6:**
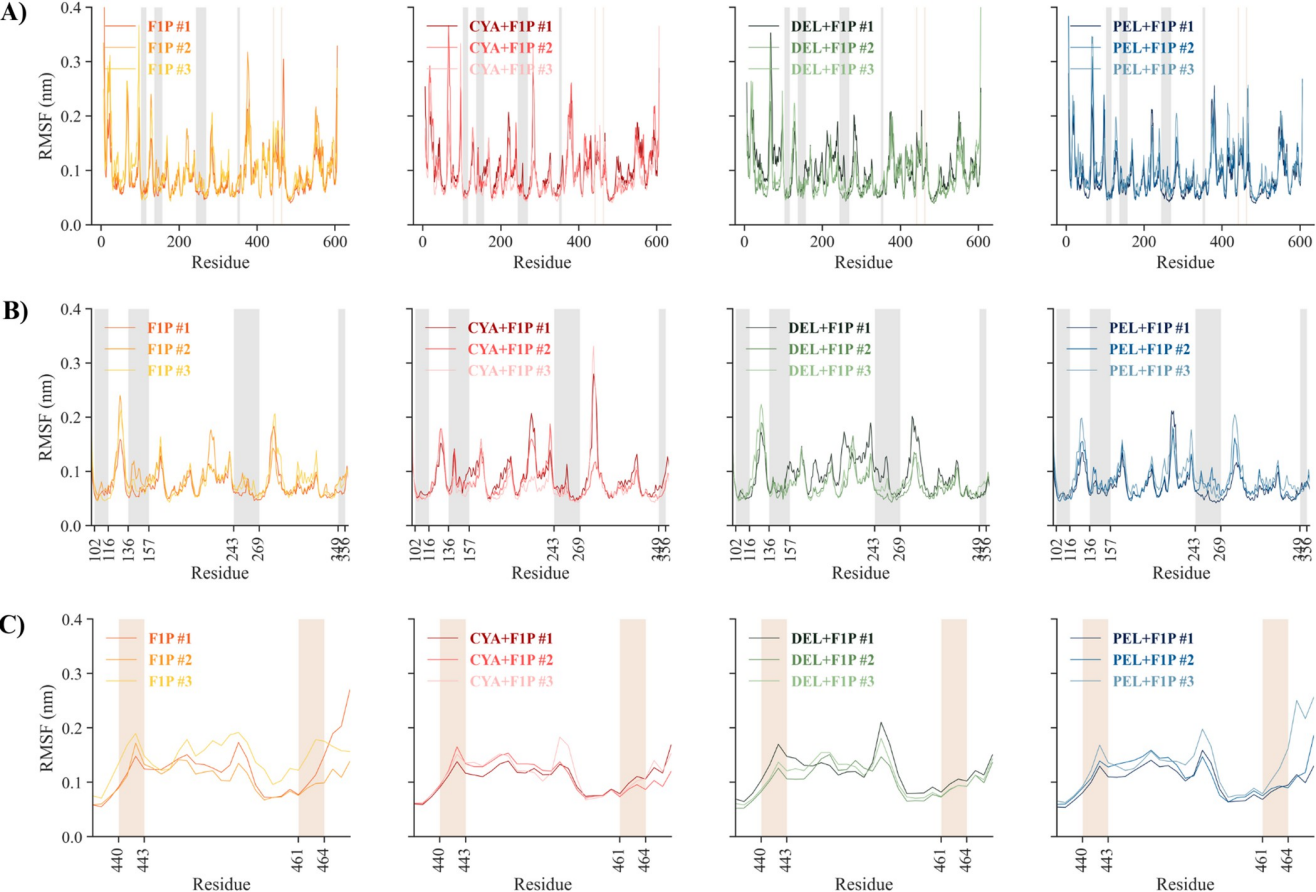
**(A-C)** RMSF graph of GKRP backbone residues in triplicates, with residues highlighted in gray and **(B)** brown consecutively indicating the crucial residues of the establishment GKRP-F1P and **(C)** GKRP-GK complex.

RMSF analysis (**Fig 6**) was also performed to describe the effect of ligand binding on the flexibility of GKRP residues. Generally, the binding of anthocyanidins with the GKRP-F1P complex results in less variability, as seen in several regions from **Fig 6A**. To determine the effect of anthocyanidins on GKRP interaction with F1P and with GK, RMSF analysis focused on specific residues that govern the interactions was also done. As depicted in **Fig 6B**, residues V102-M116, Y136-L157, F243-L269, and C349-D356 play pivotal roles in interacting with F1P [6]. Of these four regions, the regions Y136-L157 and F243-L269 differ in effect on anthocyanidin binding. Regions Y136-157 are more flexible in native GKRP-F1P and with Cya binding, while Del and Pel binding reduce the flexibility. Also, the F243-L269 region is more flexible through Cya and Del binding, while flexibility is similar for the native GKRP-F1P and Pel binding. Based on this result, the binding of anthocyanidins could affect the binding affinity of GKRP towards F1P.

On the other hand, **Fig 6C** shows residues T440-Q443 and W461-L464 that are known for their essential function for GKRP as an allosteric switch of GK through hydrophobic interaction, thereby inhibiting GK activity [38]. The binding of Cya, Del, and Pel to the GKRP-F1P complex shows no differences in T440-Q443 flexibility, while W461-L464 shows lower flexibility than the native form of GKRP-F1P complex. From this, it can be concluded that Cya, Del, and Pel can act as potential inhibitors by reducing the flexibility of the W461-L464 region, resulting in an increase in the stability of the inactive conformation of GKRP compared to GKRP’s native ligand, F1P. This resulted in the inhibition of the ability for GKRP to bind with GK, and thus, GK remained active [8].

**Fig 7:**
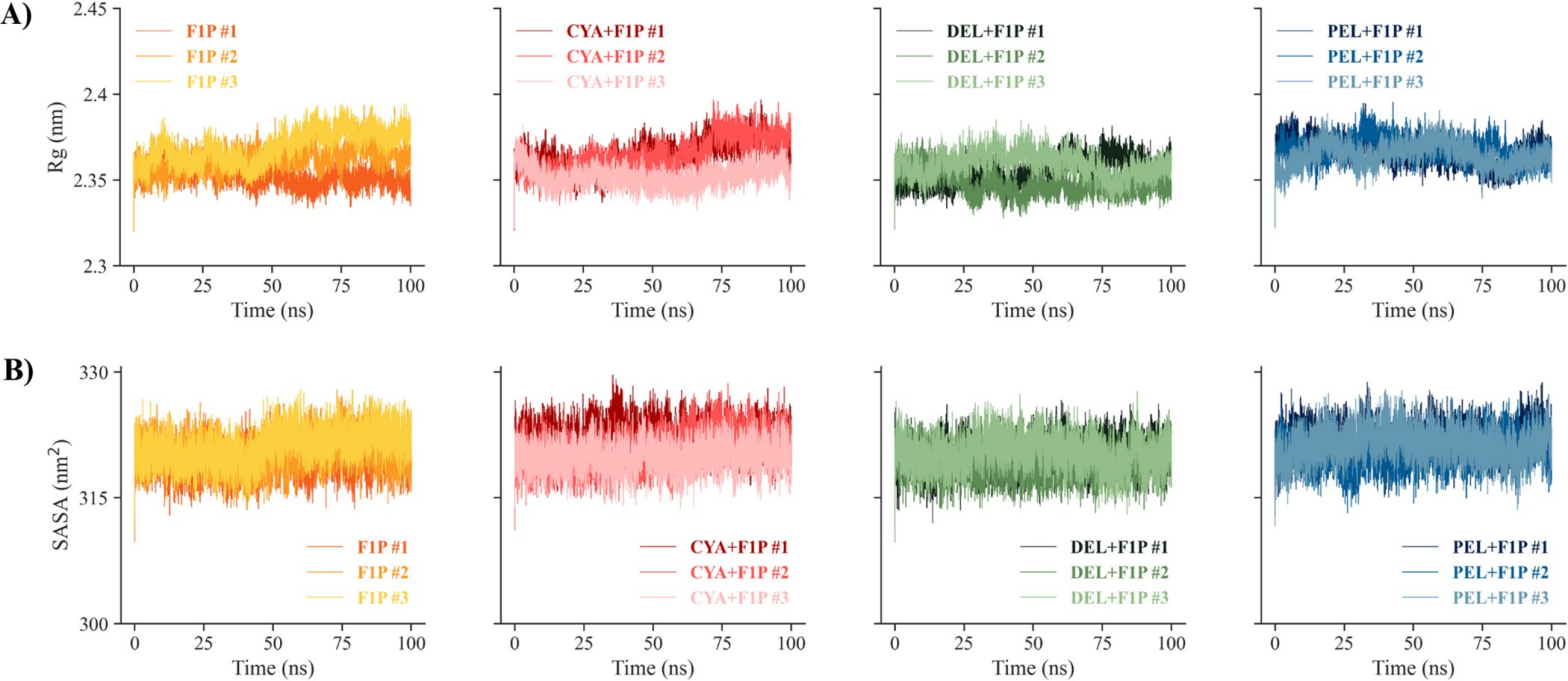
**(A)** Time-dependent Rg trajectories and **(B)** SASA dynamics of GKRP for the simulated GKRP-F1P and GKRP-F1P-anthocyanidin systems in triplicates.

The Rg points out the compactness of the examined protein structure upon ligand binding. In the presented study **(Fig 7A)**, the Rg values of native GKRP-F1P and GKRP-F1P-Cya are relatively stable, but show an increase at 50 ns and around 70 ns, respectively. This phenomenon can be explained because of its initial state of MD trajectories resulting from molecular docking, especially from the interaction between GKRP-F1P and Cya; there is an unfavourable non-bonded interaction between Cya and Ser212 of GKRP (**Fig 4C**), which leads to an increase in GKRP dynamics to find the most appropriate poses for the binding of Cya, then recalibrating its conformation to achieve global conformational stability of the GKRP-F1P-Cya complex. Compared to the SASA plot shown in **Fig 7B,** the similar trend of both figures indicates a correlation between Rg and SASA. Therefore, a relatively large SASA is present for the last 50 ns of the MD simulation for the triplicates, which correspond to variations in protein compactness in the GKRP-F1P complex **(Fig 7A)** caused by a conformational shift of the protein to a more stable structure. This is also supported by GKRP backbone RMSD pattern shown in **Fig 5A**, which was relatively stable over the last 45 ns of the simulation. Thus, relatively stable and lower Rg and SASA values of the GKRP-F1P-anthocyanidin complexes than that of the inactive GKRP-F1P form could indicate a slight increase in GKRP compactness and favor the longer binding of F1P to GKRP [6, 8].

**Fig 8:**
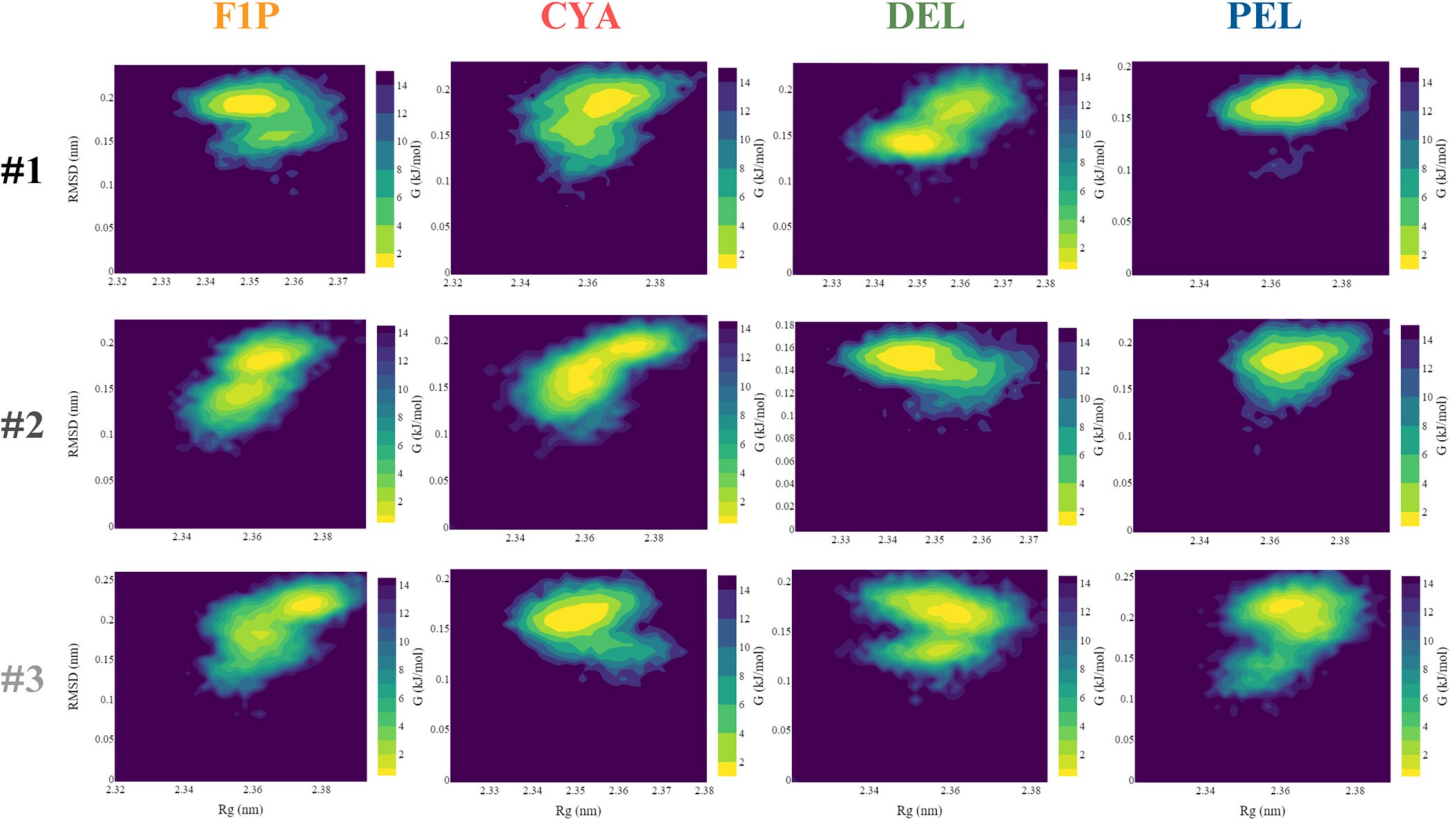
Gibbs free energy landscape of GKRP structure plotted between RMSD and Rg of 100 ns MD simulation for each system and replicas.

Furthermore, a Gibbs free energy landscape (FEL) was plotted on the GKRP backbone RMSD and Rg to evaluate the thermodynamically favourable conformational characterized by a short energy barrier between the maximum Gibbs free energy in the initial state and the global minima in the transition state [39]. The graph (**Fig 8**) shows that the energy value ranges between 0–15.6 kJ/mol, respectively for native GKRP-F1P and GKRP-F1P-anthocyanidin complexes. Based on this finding, CYA #1 to PEL #3 exhibit a large yellow-coloured basin surface area (global minima), suggesting that this complex possesses a thermodynamically stable protein structure. A larger green-coloured surface area and a small yellow-colored area (local minima) mean that the GKRP backbone visits multiple conformations before reaching the global minima. The most stable conformations of GKRP-F1P-Del from all triplicates have relatively small Rg and RMSD values, suggesting that the GKRP-F1P-Del complex is the most compact and most closely resembles the minimized GKRP structure, followed by GKRP-F1P-Cya and GKRP-F1P-Pel compared to the GKRP-F1P complex. This plot supports the former findings **(Fig 7)** regarding the effect of anthocyanidin in stabilising the GKRP-F1P complex by more compact structure such that GKRP remained inactive.

**Fig 9:**
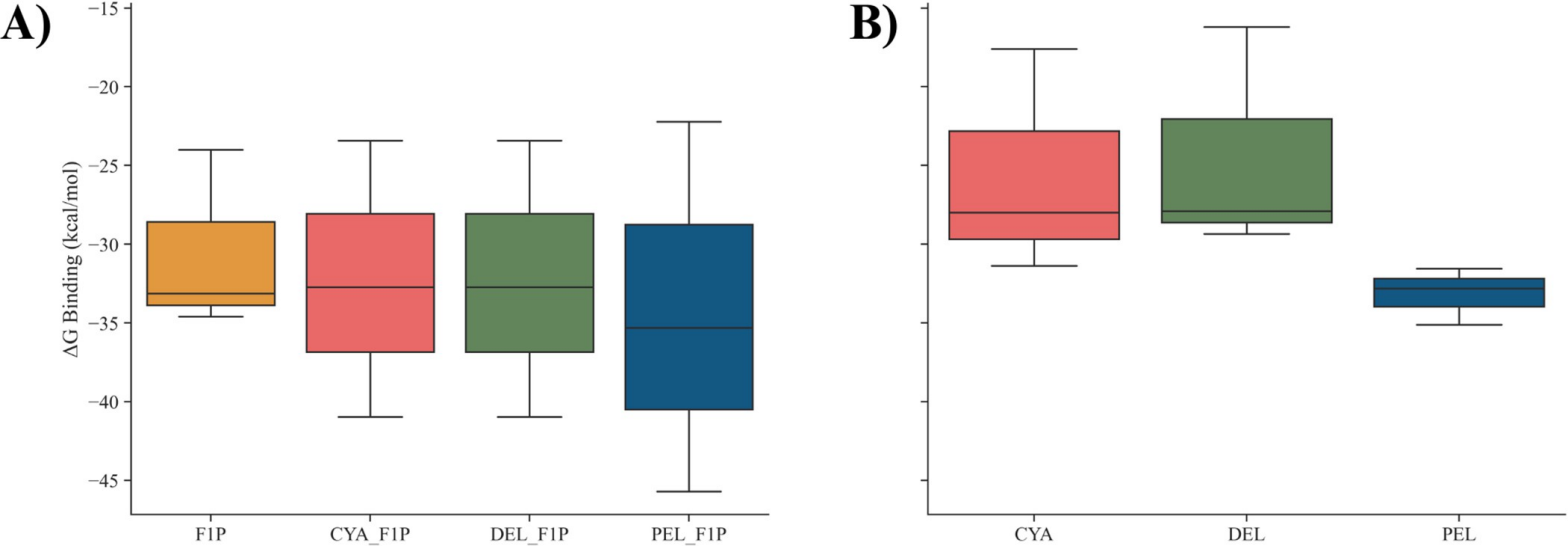
Box plot visualisation: **(A)** the MM/GBSA calculation of the binding energy of GKRP-F1P and **(B)** GKRP-anthocyanidin from the three replicates.

Binding free energy (ΔG_BIND_) is defined as the sum of all non-bonded interactions comprising of van der Waals energy (ΔE_VDW_), electrostatic energy (ΔE_ELE_), also both polar (ΔE_GB_) and non-polar (ΔE_SURF_) solvation energy. As visualised in **Fig 9**, the distribution of the triplicates binding free energies of GKRP-F1P and GKRP-F1P-anthocyanidin complexes are calculated for GKRP-F1P **(Fig 9A)** and GKRP-anthocyanidin **(Fig 9B)**. Although the GKRP-F1P binding energy variations of GKRP-F1P and GKRP-F1P-anthocyanidin are similar, as indicated by the overlapping binding energy values, it can be seen that the binding of anthocyanidin could affect the binding energy of F1P towards GKRP less than the native GKRP-F1P complex, from Pel, which has a value that has the smallest binding energy, followed by Cya and Del, which are relatively equal. From **Fig 9B**, it can be seen that the binding energy of Pel with GKRP has lower variations and lowest value, compared to Cya and Del that are similar. This demonstrates that Pel is more potential to bind to the GKRP-F1P complex and more able to decrease more the binding energy of F1P with GKRP, such that GKRP-F1P is still in the inactive form.

**Fig 10:**
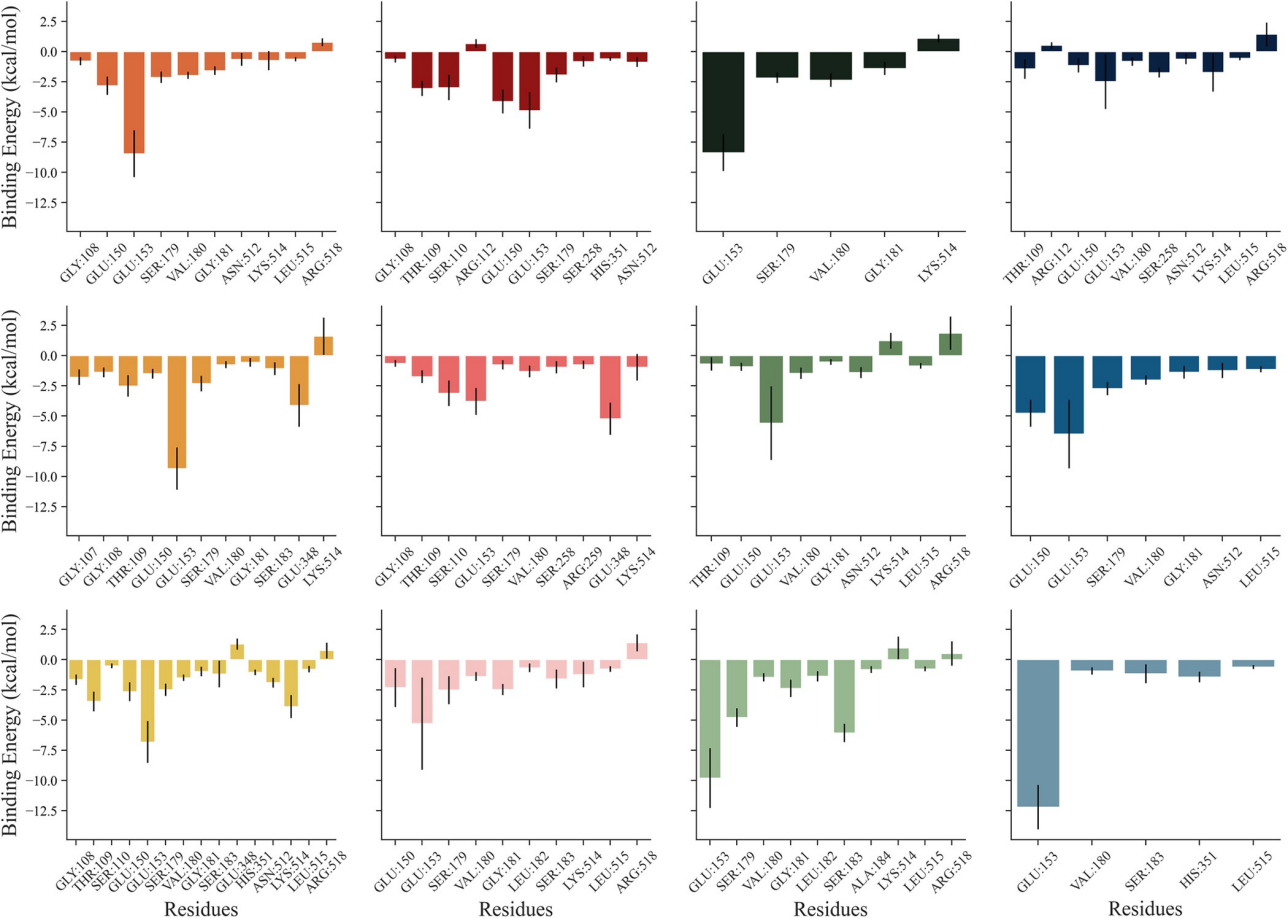
Decomposition plot (MM/GBSA) of amino acid residues within 5 Å of F1P binding site with their contributing interaction energy from each system replica. Shades of orange: F1P, shades of red: Cya, shades of green: Del, shades of blue: Pel; from above to below: #1 to #3.

In order to determine which specific residues play a pivotal role in sustaining the interactions between GKRP and the ligands, the decomposition of the binding free energy contributions from each amino acid residue was performed using the MM/GBSA method. It is evident from **Fig 10** that within 5 Å proximity of the F1P binding site, in the native form of GKRP-F1P complex, residues: Gly108, Glu150, Glu153, Ser179, Val180, Gly181, Lys514; shown in the triplicates are contributes to the successful binding of F1P to GKRP, with Glu 153 making the highest contribution, -5 –-10 kcal/mol. Binding of Cya to the GKRP-F1P complex results in a reduced contribution of Glu153 and introduce a new residue that contribute significantly to the binding process of F1P to GKRP, which is Glu348 in CYA+F1P #2. However, the binding of Del and Pel increase the binding energy contribution of Glu153 with the range of -2.5 –-12.5 kcal/mol and 0 –-15 kcal/mol, respectively. Glu153 mainly interacts with the F1P through hydrogen bonding as pictured previously in **Fig 4C**.

**Fig 11:**
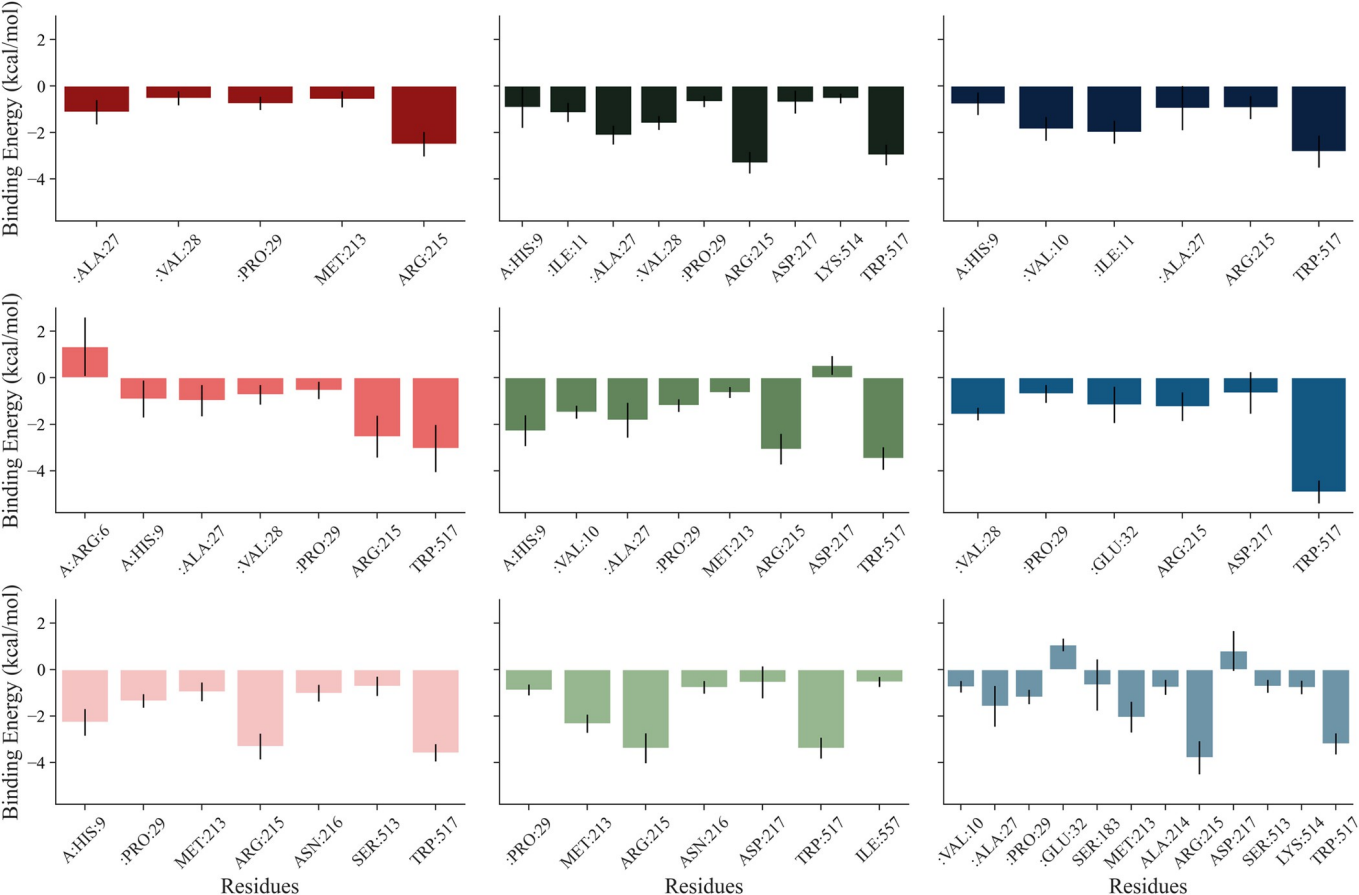
Decomposition diagram (MM/GBSA) of amino acid residues within 5 Å of anthocyanidin binding site with their contributing interaction energy from each system replica. Shades of red: Cya, shades of green: Del, shades of blue: Pel; from above to below: #1 to #3.

It further can be observed from **Fig 11** that Arg215 is the most crucial residue for the binding of anthocyanidin with GKRP-F1P complex. However, generally in Cya, Del, and Pel, Arg215 is not the main contributors of the significant binding energy, but Trp517. The presence of Trp517 which dominates the binding energy distribution among the other residues indicating that the prime interactions for the establishment of GKRP-F1P-anthocyanidin complexes are hydrophobic interactions, such as π-π stacked and π-alkyl interactions, while the Arg215, the second major contributing residue through either hydrogen bonding or hydrophobic interaction **(Fig 4C)**.

## Conclusion

The results of this research have provided a comprehensive understanding of the molecular and structural interactions between anthocyanidins and inactive GKRP-F1P form. Through quantum chemistry and molecular docking analysis, it can be seen that anthocyanidins are able to bind to GKRP and initiate various intermolecular interactions, particularly for delphinidin, cyanidin, and pelargonidin with the highest binding affinity among 6 compounds screened. Furthermore, these three compounds were subjected to molecular dynamics using GKRP-F1P as a positive control to evaluate the behaviour of GKRP-F1P complex as the effect of the binding of anthocyanidins. Based on the results of our study, the binding of pelargonidin, followed by delphinidin and cyanidin, cause an increased in the stability of GKRP backbone structure and compactness, also reduced the flexibility of residues involving in the interaction with GK, thus GKRP still established a complex between F1P remains inactive. Also, pelargonidin able to decrease larger the binding energy between F1P and GKRP, while also exhibiting the lowest binding energy among other anthocyanidins for the interaction with GKRP. Thus, in the presence of pelargonidin, GKRP will remains in the inactive conformation, allowing GK to be more functional in lowering blood glucose levels, thereby decreasing the risk of diabetes. Overall, this research provides new evidence that natural products act as antidiabetic agents by inhibiting GKRP. We hope that our findings will be continued for the *in vitro* and *in vivo* study of cyanidin and delphinidin which have these nutraceutical effects.

## Supporting information

S1 Table Ramachandran plot statistics of GKRP residues. (DOCX)

S2 Table Comparison of binding energies between Autodock Vina and QuickVina-W in anthocyanidin-GKRP complexes. (DOCX)

S1 Fig Three-dimensional visualisation of GKRP active site, indicated by green region, with blue- and red-coloured molecules are anthocyanidins and F1P (control), respectively. (DOCX)

## Abbreviations

ACPYPE: AnteChamber PYthon Parser interfacE
B3LYP: Becke, 3-parameter, Lee-Yang-Parr
F1P, 1-O: -phosphono-beta-D-fructopyranose
GAFF2: General AMBER Force Field 2
G6P: glucose 6-phosphate
HOMO: Highest Occupied Molecular Orbital
LUMO: Lowest Unoccupied Molecular Orbital
MM/GBSA: Molecular Mechanics with Generalized Born Surface Area
NPT: Constant Number-Pressure-Temperature
NVT: Constant Number-Volume-Temperature
