# Supplementary material for: Molecular insights into the inhibitory potential of anthocyanidins on glucokinase regulatory protein
Source: PLoS One. 2023 Jul 19;18(7):e0288810. doi: 10.1371/journal.pone.0288810 (PMC10355436; doi:10.1371/journal.pone.0288810)
Supplement: S1 Table — (DOCX) [file pone.0288810.s001.docx]

**S1 Table. Ramachandran plot statistics of GKRP residues.**

| **Ramachandran Plot** | **Before EM** | **After Gromacs-EM** | **After Yasara-EM** |
| --- | --- | --- | --- |
| Favored | 91.4% | 89.3% | 92.9% |
| Allowed | 8.5% | 10.5% | 6.9% |
| Outliers | 0.2% | 0.2% | 0.2% |
