# Supplementary material for: Molecular insights into the inhibitory potential of anthocyanidins on glucokinase regulatory protein
Source: PLoS One. 2023 Jul 19;18(7):e0288810. doi: 10.1371/journal.pone.0288810 (PMC10355436; doi:10.1371/journal.pone.0288810)
Supplement: S2 Table — (DOCX) [file pone.0288810.s002.docx]

**S2 Table. Binding energy comparison of AutodockVina and QuickVina-W in Anthocyanidin-GKRP complexes.**

|  | **Binding Energy (BE, kcal/mol)** | | | | | |
| --- | --- | --- | --- | --- | --- | --- |
|  | **Cya-GKRP** | **Del-GKRP** | **Mal-GKRP** | **Pel-GKRP** | **Peo-GKRP** | **Pet-GKRP** |
| **AutodockVina** | -8.36 $\pm$ 0.05^b^ | -8.54 $\pm$ 0.07^b^ | -8.13 $\pm$ 0.04^b^ | -8.15 $\pm$ 0.06^b^ | -7.99 $\pm$ 0.03^b^ | -7.84 $\pm$ 0.13^b^ |
| **QuickVina-W** | -8.6^a^ | -8.7^a^ | -8.2^a^ | -8.6^a^ | -8.4^a^ | -8.38 $\pm$ 0.04^a^ |

The data was collected in quintuplicate. The values are expressed as mean ± standard deviation (SD). Different letters within the same column indicate statistically significant differences (p<0.05), determined by Mann-Whitney test.
