# Supplementary material for: Molecular insights into the inhibitory potential of anthocyanidins on glucokinase regulatory protein
Source: PLoS One. 2023 Jul 19;18(7):e0288810. doi: 10.1371/journal.pone.0288810 (PMC10355436; doi:10.1371/journal.pone.0288810)
Supplement: S1 Fig — (DOCX) [file pone.0288810.s003.docx]

**
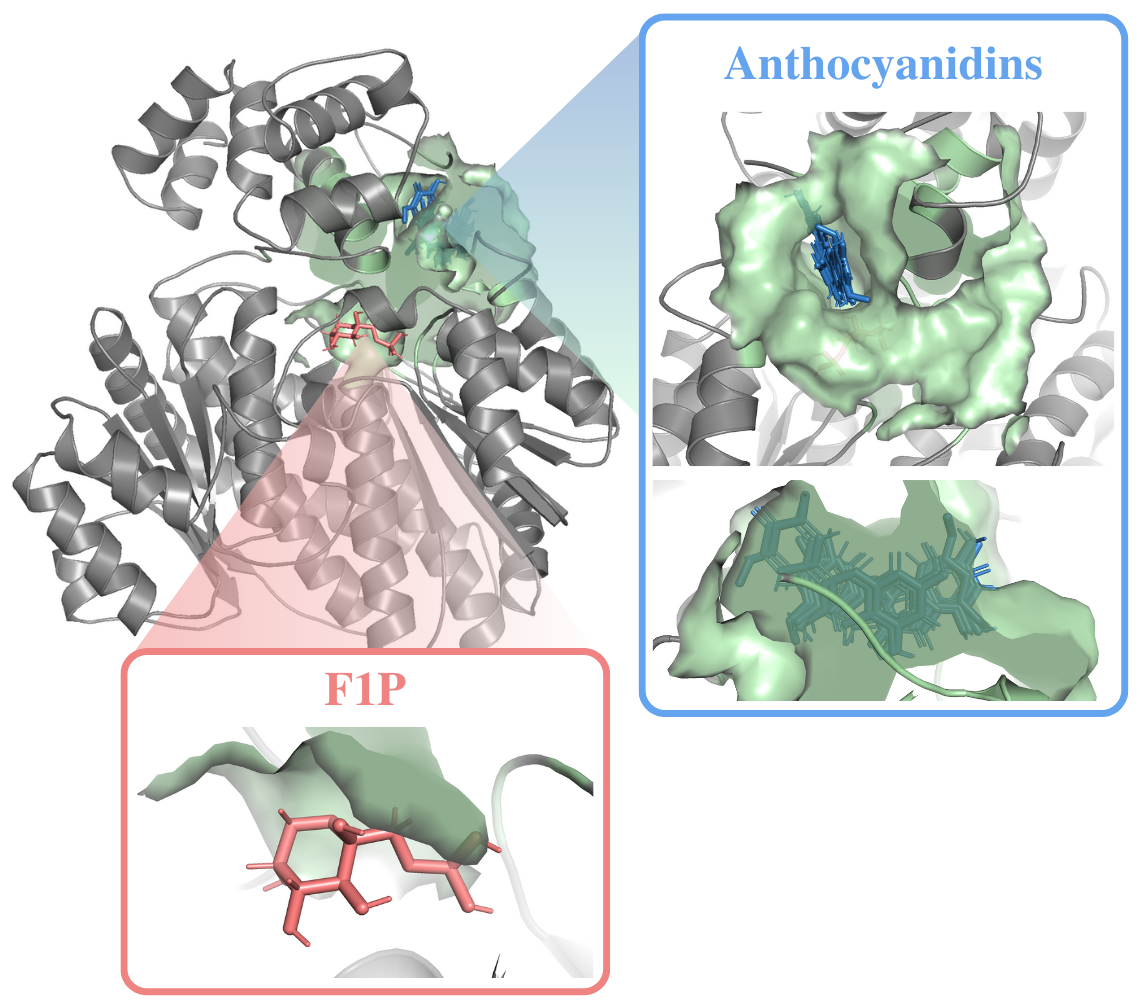
**

**S1 Fig. Three-dimensional visualisation of GKRP active site, indicated by green region, with blue- and red-coloured molecules are anthocyanidins and F1P (control), respectively.**
